# Emetine inhibits Zika and Ebola virus infections through two molecular mechanisms: inhibiting viral replication and decreasing viral entry

**DOI:** 10.1038/s41421-018-0034-1

**Published:** 2018-06-05

**Authors:** Shu Yang, Miao Xu, Emily M Lee, Kirill Gorshkov, Sergey A. Shiryaev, Shihua He, Wei Sun, Yu-Shan Cheng, Xin Hu, Anil Mathew Tharappel, Billy Lu, Antonella Pinto, Chen Farhy, Chun-Teng Huang, Zirui Zhang, Wenjun Zhu, Yuying Wu, Yi Zhou, Guang Song, Heng Zhu, Khalida Shamim, Carles Martínez-Romero, Adolfo García-Sastre, Richard A. Preston, Dushyantha T. Jayaweera, Ruili Huang, Wenwei Huang, Menghang Xia, Anton Simeonov, Guoli Ming, Xiangguo Qiu, Alexey V. Terskikh, Hengli Tang, Hongjun Song, Wei Zheng

**Affiliations:** 1 National Center for Advancing Translational Sciences, National Institutes of Health, 9800 Medical Center Drive, Bethesda, MD 20892 USA; 2 Department of Biological Science, Florida State University, Tallahassee, FL 32306 USA; 3 Development, Aging and Regeneration Program, Sanford Burnham Prebys Medical Discovery Institute, La Jolla, CA 92037 USA; 4 Special Pathogens Program, National Microbiology Laboratory, Public Health Agency of Canada, Winnipeg, Manitoba R3E 3R2 Canada; 5 Department of Biomedical Sciences, Florida State University, Tallahassee, FL 32306 USA; 6 Department of Pharmacology & Molecular Sciences, Johns Hopkins School of Medicine, Baltimore, MD USA; 7 Department of Microbiology and Global Health and Emerging Pathogens Institute, Icahn School of Medicine at Mount Sinai, New York, NY 10029 USA; 8 Department of Medicine, Miller School of Medicine, University of Miami, Miami, FL 33136 USA; 9 Department of Neuroscience and Mahoney Institute for Neurosciences, University of Pennsylvania, Philadelphia, PA 19104 USA; 10 Department of Medical Microbiology, University of Manitoba, Winnipeg, Manitoba R3E 0J9 Canada

## Abstract

The re-emergence of Zika virus (ZIKV) and Ebola virus (EBOV) poses serious and continued threats to the global public health. Effective therapeutics for these maladies is an unmet need. Here, we show that emetine, an anti-protozoal agent, potently inhibits ZIKV and EBOV infection with a low nanomolar half maximal inhibitory concentration (IC_50_) in vitro and potent activity in vivo. Two mechanisms of action for emetine are identified: the inhibition of ZIKV NS5 polymerase activity and disruption of lysosomal function. Emetine also inhibits EBOV entry. Cephaeline, a desmethyl analog of emetine, which may be better tolerated in patients than emetine, exhibits a similar efficacy against both ZIKV and EBOV infections. Hence, emetine and cephaeline offer pharmaceutical therapies against both ZIKV and EBOV infection.

## Introduction

The recent spread of Zika virus (ZIKV) infection to the Americas in 2015 and 2016 has quickly motivated research into ZIKV disease pathogenesis and development of therapeutic treatments^1^. Prior to its spread to the Western Hemisphere, ZIKV disease was classified as a mild, self-limiting febrile illness in ~20% of ZIKV infected individuals with the remainder of patients being asymptomatic. However, the recent 2015–2016 outbreaks of ZIKV in the Americas have been associated with complications including microcephaly, neurodevelopmental disorders, and Guillain–Barré syndrome, precipitating the need for development of effective drug therapies^2^.

A year prior to the America’s ZIKV outbreak, Ebola virus (EBOV) re-emerged in a deadly viral outbreak in West Africa during 2014 through 2016 with a 50% mortality rate with 11,310 confirmed deaths^3^. Although research has identified several Food and Drug Administration (FDA)-approved drugs with activities against ZIKV and EBOV infections^4–7^, there are still no anti-viral drugs approved by the FDA specifically for the treatment of EBOV or ZIKV.

We have recently employed a high-throughput assay using a pair of anti-ZIKV non-structural protein 1 (NS1) antibodies for a ZIKV drug repurposing screen^8^. One of the compounds identified in the screen was the alkaloid emetine. Emetine’s structural desmethyl analog is cephaeline. Emetine, an FDA-approved compound for amoebiasis, has previously demonstrated anti-viral activity against other viruses^9^, but its inhibitory effects and mechanism of action in ZIKV are unknown. Here, we investigated emetine and cephaeline in the context of ZIKV and EBOV infection to uncover their effects on both viral machinery and host cell interactions using in vitro and in vivo models.

## Results

### Emetine potently inhibits ZIKV replication in vitro

**Fig. 1:**
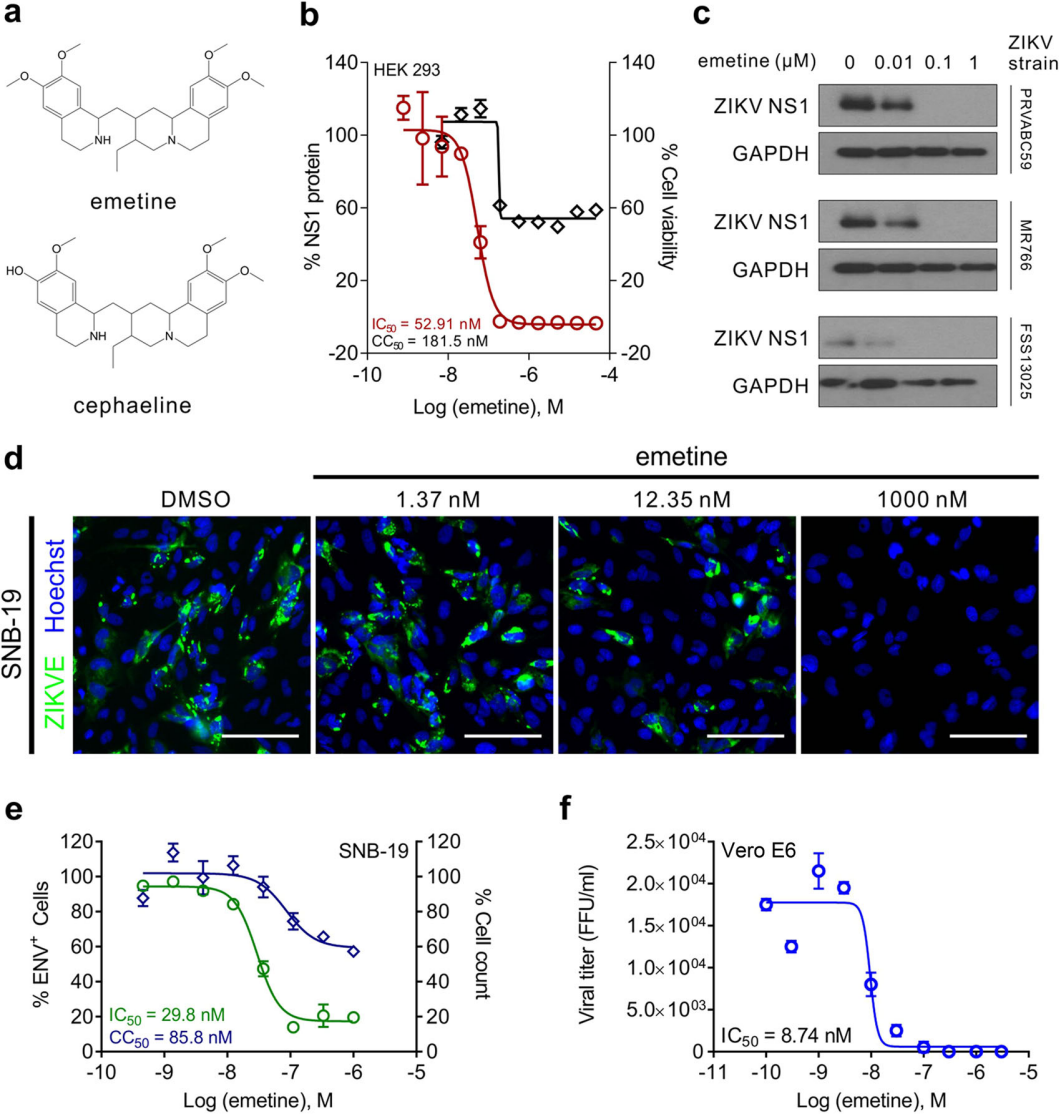
Emetine is an inhibitor of ZIKV infection and replication. **a** Chemical structures of emetine and cephaeline. **b** Dose-response curve showing the effect of emetine treatment on ZIKV NS1 protein expression (red) and cell viability (black) in HEK293 cells exposed to ZIKV MR766 strain. **c** Western blot of ZIKV NS1 protein after treatment of SNB-19 cells with varying concentrations of Emetine. SNB-19 cells were treated with emetine for 1 h before inoculation with ZIKV strains PRVABC59, FSS13025, or MR766 (MOI = 1), and cells were harvested 24 h after infection for Western Blot analysis. **d** Immunofluorescent images of astrocytoma cells stained for ZIKV PRVABC59 envelope (ENV) protein (green), nuclei (blue) and treated with emetine. Scale bar=100 µm. **e** Dose–response curves from cells in (**d**) showing the inhibition effect of emetine on ZIKV PRVABC59 infection measured by ENV+ SNB-19 cells (green), and cell number by nuclear counting (blue). **f** SNB-19 cells were treated with emetine at increasing concentrations for 1 h before infection with PRVABC59 at a MOI = 1 (*n* = 3 cultures). Cell culture supernatant was collected 24 h after infection, and infectious virions were quantified for focus-forming units (FFU) using Vero cells. All curves represent best fits for calculating the IC_50_ values (graph inset). All values represent mean ± SD (*N* = 3 replicates)

By measuring secreted ZIKV-NS1 protein, a marker of ZIKV infection and replication in host cells, we identified the lead compound emetine, an anti-protozoal agent (Fig. 1a), and its analog cephaeline, in a drug repurposing screen^8^. We first found that emetine dose-dependently decreased NS1 protein level in HEK293 cells infected with the African prototype, ZIKV MR766 (IC_50_ = 52.9 nM; 95% confidence interval (CI) of 35.4–73.2 nM) (Fig. 1b). To validate that the effect was not viral strain specific, we also tested the effect of emetine on ZIKV NS1 protein expression in human glioblastoma SNB-19 cells infected with three different ZIKV isolates PRVABC59 (2016 Puerto Rico isolate), the previously-mentioned MR766, and the 2010 Cambodian isolate FSS13025, as measured by Western blot (Fig. 1c). In an immunofluorescence staining assay using the anti-ZIKV envelope (ENV) protein antibody, emetine inhibited PRVABC59 (Fig. 1d, e) and MR766 (Supplementary Figure S1) E-protein expression in SNB-19 cells (IC_50_ = 29.8 nM with CI of 24.4–35.0 nM), further confirming emetine’s activity against ZIKV replication. In the classical ZIKV titer assay using Vero cells, emetine completely suppressed viral replication (IC_50_ = 8.74 nM with CI of 7.4–10.7 nM) (Fig. 1f and Supplementary Figure S1b-c). With emetine or dimethyl sulfoxide (DMSO) treatment prior to ZIKV infection, ZIKV RNA levels were comparable at time-points corresponding to binding and initial entry and translation, with initial differences observed around 8 h post infection (Supplementary Figure S1d). While emetine partially suppressed HEK293 cell viability and SNB-19 cell growth in the absence of the virus (half maximal cytotoxic concentration (CC_50_) = 180 nM and 86 nM, respectively), this effect was seen at more than 10-fold the IC_50_ necessary for viral inhibition, indicating that its antiviral effect is independent of cytotoxicity (Fig. 1b and Supplementary Figure S1e-f). In addition, cephaeline, a desmethyl analog of emetine, similarly inhibited ZIKV replication (Supplementary Figure S2a-c). These results indicate that emetine is a potent inhibitor of ZIKV replication in vitro. The inhibitory effect is seen in SNB-19, HEK293, and Vero E6 cells, demonstrating that the effect is not cell line specific.

In order to understand the stage of the ZIKV life cycle affected by emetine and cephaeline treatment, we varied compound treatment before, during, and after ZIKV inoculation (Supplementary Figure S2d). Emetine and cephaeline exhibited low nanomolar IC_50_ values of less than 42 nM for the three groups where cells had extended exposure to the drug. Together, these data indicate that the antiviral effect is most likely post-entry and at the step of viral replication.

### Emetine inhibits ZIKV NS5 polymerase activity

**Fig. 2:**
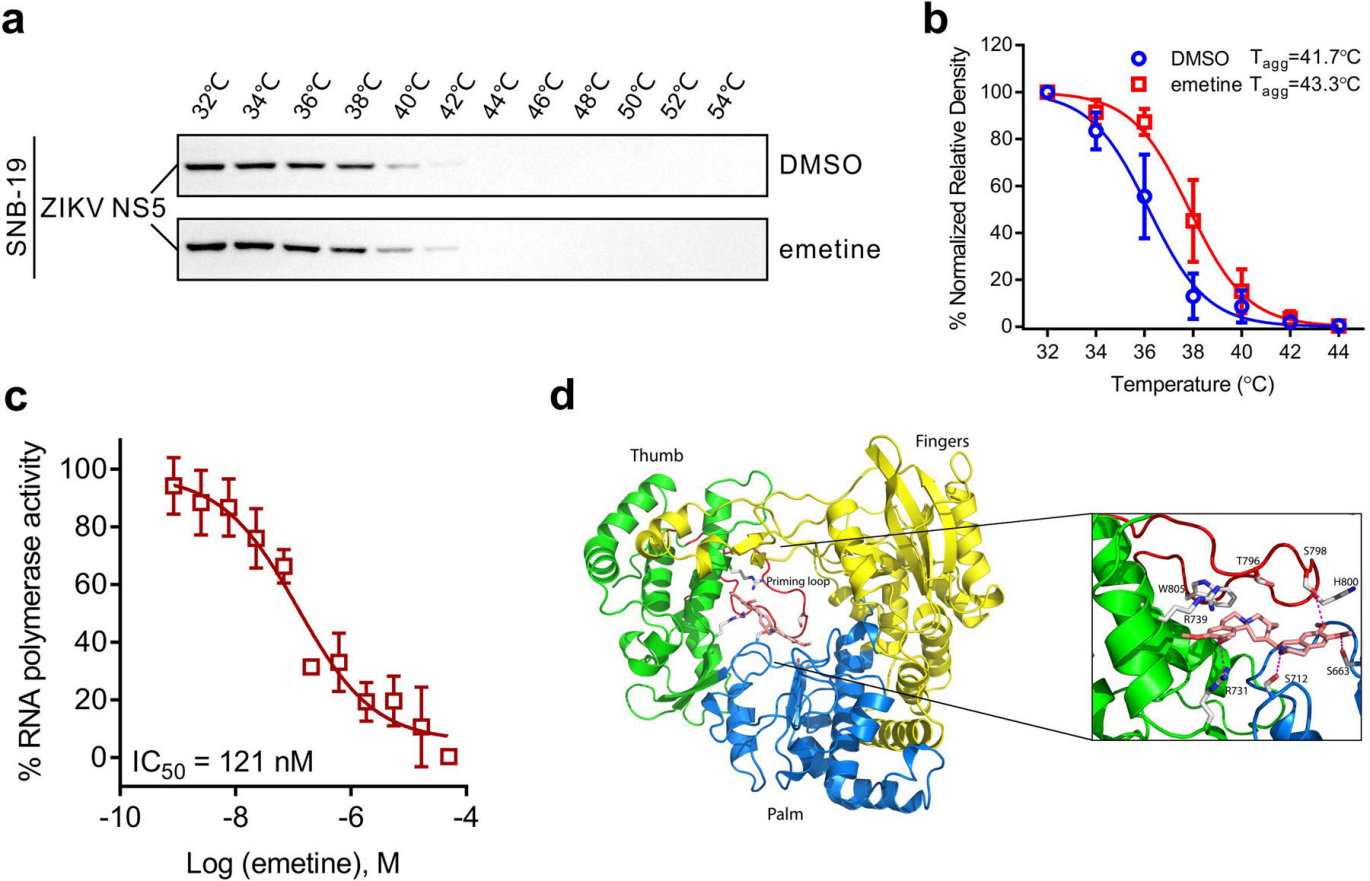
Emetine inhibits ZIKV NS5 polymerase activity. **a** Western blot showed the CETSA binding assay of ZIKV NS5 in the presence or absence of 50 μM emetine at different temperatures. **b** Temperature melting curves of ZIKV NS5 in (**c**). The relative chemiluminescent intensity of each sample at different temperatures was used to generate temperature-dependent melting curves and the apparent aggregation temperature (T_agg_) was calculated by nonlinear regression. Values represent mean ± SEM (*N* = 3 replicates). **c** Dose–response curve showing the inhibition effect of emetine treatment on the RNA-dependent RNA polymerase (RdRp) activity of recombinant ZIKV NS5 enzyme. The curve represent best fits for calculating the IC_50_ values (graph inset). Values represent mean ± SD (*N* = 3 replicates). **d** Predicted binding model of emetine with ZIKV NS5 RNA-dependent RNA-polymerase (RdRp). The three domains of fingers (yellow), palm (blue), and thumb (green) are shown in ribbons, while the priming loop is shown in red color. The emetine bound at the active site is depicted as sticks. A close-up view of hydrogen-binding interactions of emetine at the active site of RdRp is shown

The data presented thus far suggest that emetine acts to inhibit viral replication. To determine emetine’s mechanism of action, we examined if it could bind to and inhibit the function of the ZIKV NS5 (non-structural protein 5) RNA-dependent RNA polymerase (RdRp). First, we employed a cellular thermal shift assay (CETSA) that quantifies the amount of stabilized protein after thermal unfolding and aggregation. Values of apparent aggregation temperature (T_agg_) of NS5 in ZIKV-infected SNB-19 cell lysates were measured in the absence or presence of emetine at temperatures ranging from 32 to 54 °C (Fig. 2a). The best-fit curve from the emetine treated group was significantly different with the DMSO control (*p* < 0.001), and emetine markedly increased the T_agg_ of ZIKV NS5 protein from 41.7 to 43.3 °C (Fig. 2b), suggesting emetine bound directly to ZIKV NS5 and protected it from thermallly induced aggregation.

To further explore if emetine inhibited ZIKV NS5 polymerase activity, we performed a cell-free recombinant ZIKV NS5 assay and found that emetine directly inhibited ZIKV NS5 RNA polymerase activity with an IC_50_ of 121 nM (CI of 64 to 258 nM) (Fig. 2c). Molecular modeling and docking studies further showed the structural basis of emetine binding interactions with the ZIKV NS5 polymerase (Fig. 2d). The predicted binding model revealed that emetine preferably bound to an allosteric site adjacent to the priming loop, the “N pocket”, as observed in dengue virus RNA-dependent RNA polymerase (RdRp) for non-nucleoside inhibitor (NNI) binding^10,11^, where emetine forms H-bonding and aromatic stacking interactions with several catalytic essential residues (Supplementary Figure S3a). Similarly, cephaeline inhibited ZIKV NS5 RdRp in the polymerase activity assay (Supplementary Figure S3b). Together, the results indicated that emetine potently inhibits ZIKV NS5 RNA polymerase activity by binding to its active site.

### Emetine suppressed ZIKV load in vivo

**Fig. 3:**
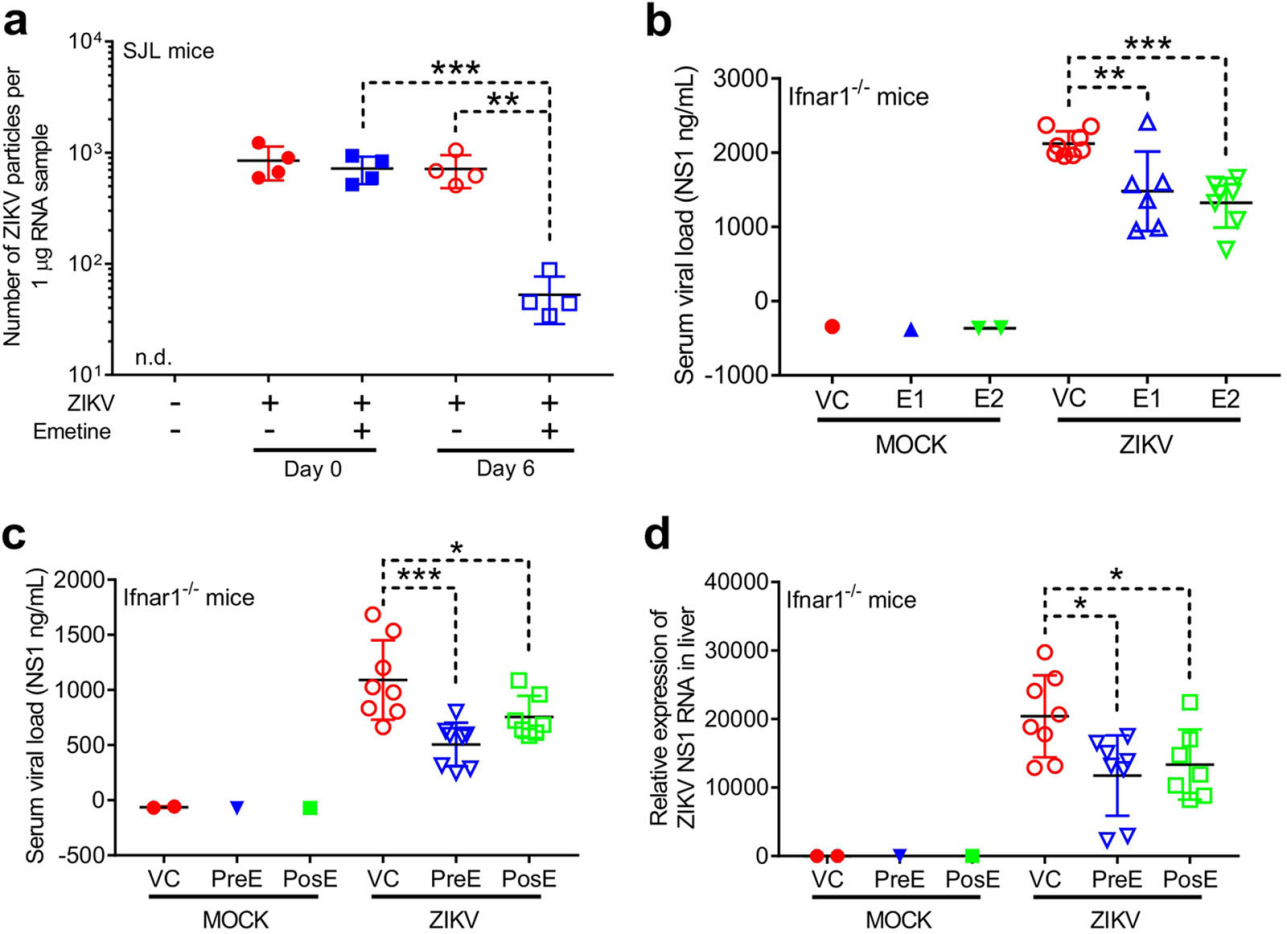
Emetine suppresses ZIKV virus load in vivo. **a** Three-month-old SJL male mice were infected retro-orbitally with ZIKV^BR^ followed by IP administration of emetine (1 mg/kg/day) for the next 6 days (*N* = 4 mice per group). Two groups of SJL mice (*N* = 4) received the same volume of vehicle buffers. On the day 7 the blood samples were collected and ZIKV RNA was quantified by real-time qPCR. Statistical analysis by two tailed *t*-test. **p = 0.0014, ***p = 0.0005. **b** Ifnar1^−/−^^−/−^ mice were dosed with emetine 1 mg/kg (E1, *N* = 6), 2 mg/kg (E2, *N* = 7), and PBS (VC, *N* = 8), respectively, 24 h prior to challenge with virus on day 0. Treatment with emetine/VC was done once daily intraperitoneally. On day 3, a blood sample was taken from tail vein and viral load in the serum is estimated by ZIKV NS1 ELISA kit. Values presented as mean ± SEM, one way ANOVA, followed by Dunnett’s test. **p = 0.007, ***p = 0.0008. **c** Ifnar1^-/-^ mice (8–9 week old, male and female) were dosed with emetine 2 mg/kg/day IP starting at 24 h prior (*N* = 8) or 24 h after (*N* = 7) challenging with ZIKV on day 0. Drug was continued until day 3 and mice were killed. Blood was collected and the serum NS1 protein was measured using ZIKV NS1 ELISA kit. **d** Liver tissue sample was collected, total RNA was column-extracted and qPCR was done using ZIKV NS1 specific primers and GAPDH primers to calculate the relative expression of the NS1 gene. Statistical analysis was done using the Mann–Whitney ANOVA. *p < 0.05, ***p < 0.001

Next, we tested whether emetine could reduce ZIKV infection in two distinct mouse models. In the first well-characterized model for ZIKV infection^12,13^, we inoculated immunocompetent three-month-old female SJL mice with ZIKV^BR^ (1 × 10^5^ plaque forming unit (PFU); Brazilian strain, Brazil-ZIKV2015) and delivered emetine via retro-orbital injection at 1 mg/kg/day for six days. The level of ZIKV infection was determined by qPCR analysis of blood plasma. The viremia profiles for the SJL mouse model have previously determined that the infection is stable from 6 d.p.i to 3 months post-infection^14^. Emetine treatment reduced the levels of circulating ZIKV approximately 10-fold (Fig. 3a). In the second model, severely immunocompromised Ifnar1^−/−^ mice were infected with ZIKV FSS13025 via intraperitoneal (IP) injection. We analyzed the ZIKV viremia profile in Ifnar1^−/−^ mice and found peak infection between 3 and 5 d.p.i (Supplementary Figure S4a). The serum viral load of ZIKV infected Ifnar1^−/−^ mice was significantly lower when animals were treated with emetine compared with vehicle control (VC) (Fig. 3b). Furthermore, the NS1 protein and ZIKV RNA in Ifnar1^−/−^ mice treated with 2 mg/kg/day emetine pre or post-ZIKV infection were significantly decreased in serum and liver, respectively (Fig. 3c, d), as well as cephaeline treatment (Supplementary Figure S4b). Altogether, these data indicate that emetine is a potent suppressor of ZIKV infection in vivo.

### Emetine inhibited EBOV infection in vitro and in vivo

**Fig. 4:**
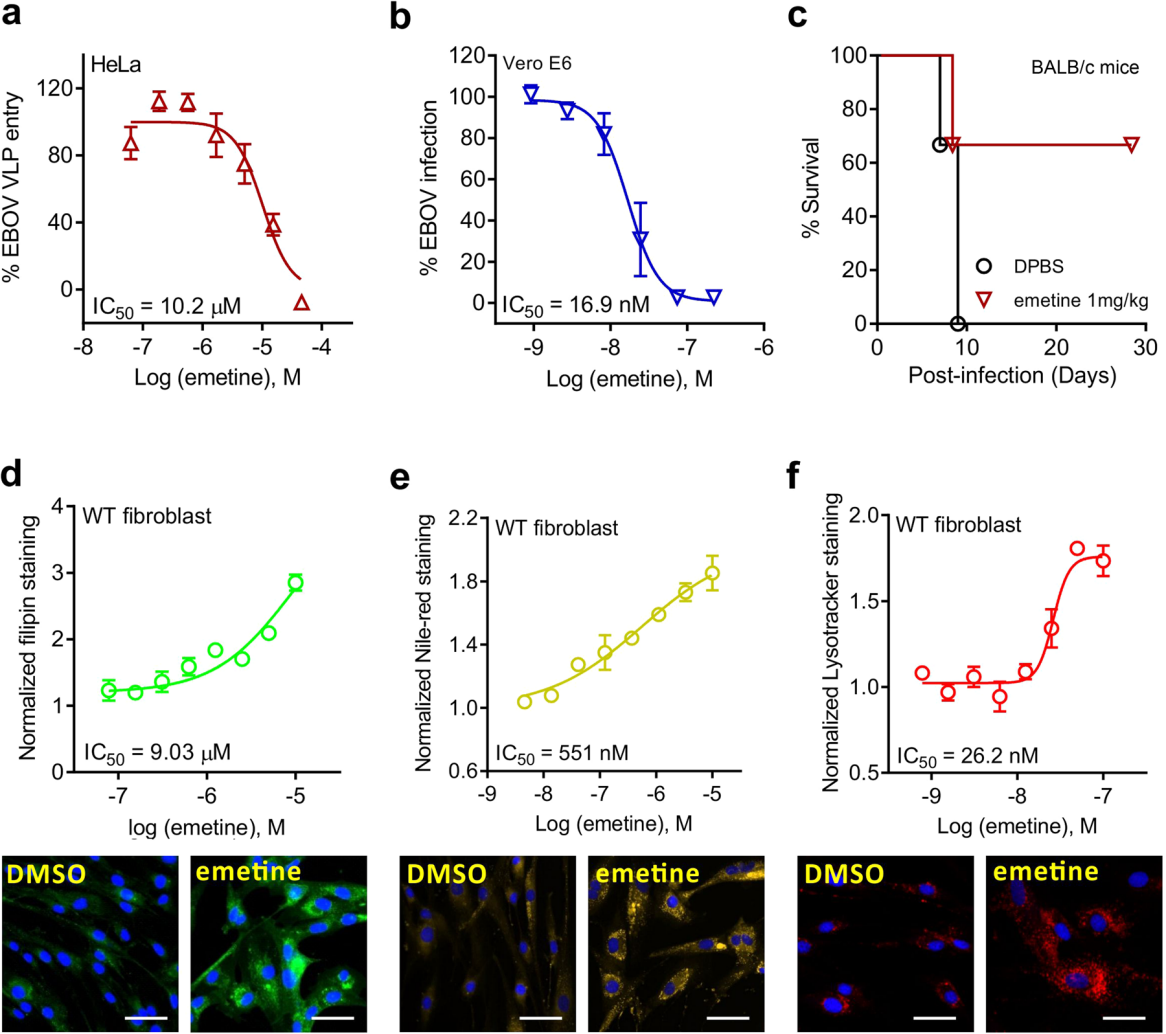
Emetine inhibits EBOV infection in vitro and in vivo. **a** Dose–response curve showing the inhibition effect of emetine treatment on Ebola VLP entry in HeLa cells. **b** Dose–response curve showing the inhibition effect of emetine treatment on infection of Ebola live virus in Vero E6 cells. **c** The survival curve of MA-EBOV infected mouse treated with 1 mg/kg emetine every day. Six to eight week-old female BALB/c mice were randomly assigned into groups (*N* = 6 animals). All the mice were challenged with a lethal dose of 1000 times the LD_50_ mouse adapted EBOV via IP treatments with either emetine (1 mg/kg/day) or PBS (same volume for the control group) were initiated at 3 h before the challenge and continued for up to 6 days post infection. Survival was monitored for 28 days post infection. **d** Top, dose–response curve showing the effect of emetine on cholesterol accumulation measured by filipin dye fluorescence intensity (IC_50_ = 9.03 µM). Bottom, fluorescence images of fibroblast cells treated with DMSO or emetine and stained with filipin (unesterified free cholesterol, green) and nuclear green (nuclei, blue). **e** Top, dose–response curve showing the effect of emetine on lipid accumulation measured by Nile red fluorescence intensity (IC_50_ = 551 nM). Bottom, fluorescence images of fibroblast cells treated with DMSO or emetine and stained with Nile red (lipids, yellow) and Hoechst 33342 (nuclei, blue). **f** Top, dose–response curve showing the effect of emetine on acidic organelle accumulation measured by LysoTracker dye fluorescence intensity (IC_50_ = 26.2 nM). Bottom, fluorescence images of fibroblast cells treated with DMSO or emetine and stained with LysoTracker dye (acidic organelles, red) and Hoechst 33342 (nuclei, blue). All curves represent best fits for calculating the IC_50_ values (graph inset). All values represent mean ± SD (*N* = 3 replicates). Scale bar=50 µm

Emetine was previously reported to inhibit EBOV polymerase activity determined in a minigenome assay but its potency and mechanism of action were unknown^15,16^. To determine whether emetine blocks EBOV entry, we employed a previously optimized Ebola viral like particle (VLP) entry assay^17^ and found a dose-dependent inhibition of EBOV VLP entry into HeLa cells (IC_50_ = 10.2 μM with 95% CI of 7.22–14.4 μM) (Fig. 4a). Importantly, we confirmed emetine’s activity against EBOV infection in the live virus assay using Vero E6 cells (IC_50_ = 16.9 nM with 95% CI of 10.7–25.8 nM) (Fig. 4b and Supplementary Figure S5a). Cephaeline exhibited similar activity against EBOV entry and replication in vitro (Supplementary Figure S5b-d). Therefore, emetine and cephaeline exhibited potent inhibition of both ZIKV and EBOV infections in vitro.

We next tested the protective efficacy of Emetine emetine in an EBOV mouse model. Six to eight week-old, female BALB/c mice (*n* = 6 per group) were injected IP with 1000-times the mean lethal dose for 50% (LD_50_) of mouse-adapted Ebola virus (MA-EBOV). Before infection with MA-EBOV, mice were then treated either with emetine (1 mg/kg/day), cephaeline (5 mg/kg/day) or VC (in the control group) starting 3 h before viral innoculation via IP. After IP administration of MA-EBOV, mice continued treatment with emetine, cephaeline, or VC IP for 7 more days. The animals were monitored daily for survival. As expected, all of the control animals uniformly succumbed to EBOV infection with a mean time to death of 8.33 ± 1.03 d.p.i. In contrast, for 67%, or four out of six mice, survival was achieved in both treated groups (Fig. 4c and Supplementary Figure S5e-f). Similar to the effects of emetine and cephaeline treatment in ZIKV infection, the drugs effectively suppressed EBOV infection in vivo.

### Emetine accumulates in lysosomes and inhibits their function leading to decreased viral entry

Given that cellular cholesterol hemostasis and autophagy were reported to be a critical factor for virus entry and replication, we next examined emetine’s effect on cholesterol and lysosome accumulation^18–20^. Three fluorescence dye staining assays were employed. The filipin dye staining assay measures unesterified cholesterol accumulation in lysosomes, indicating inhibition or deficiency of cholesterol transport function^21^. The Nile red dye staining assay detects accumulation of nonpolar lipids in lysosomes, another indicator of lysosomal dysfunction^21^. The LysoTracker dye detects enlarged acidic organelles (lysosomes and late endosomes) due to accumulation of lipids/macromolecules, indicating deficient functions in lysosomes and late endosomes^22^. Emetine and cephaeline dose-dependently increased lysosomal staining of filipin in fibroblasts (Fig. 4d and Supplementary Figure S6a). An increase in Nile red in the presence of emetine was also seen in fibroblasts (Fig. 4e and Supplementary Figure S6b). Finally, LysoTracker dye staining was also increased in fibroblasts treated with emetine (Fig. 4f and Supplementary Figure S6c). The results indicated that emetine and cephaeline led to an accumulation of cholesterol, probably due to an alteration in the pH of the lysosomes and an impairment in intracellular traffic of these organelles.

Interestingly, based on its chemical property as a weak base, emetine may accumulate with high concentrations in lysosomes leading to inhibition of lysosomal function. To test this idea, we labeled cephaeline with boron-dipyrromethene (BODIPY), a dye used to tag low molecular weight molecules for tracking inside of cell. We found that this labeled probe entered cells quickly and accumulated in lysosomes (Supplementary Figure S6d). Altogether, these results suggest that in lysosomes a locally high concentration of cephaeline contributes to its potent activity against EBOV infections.

**Fig. 5:**
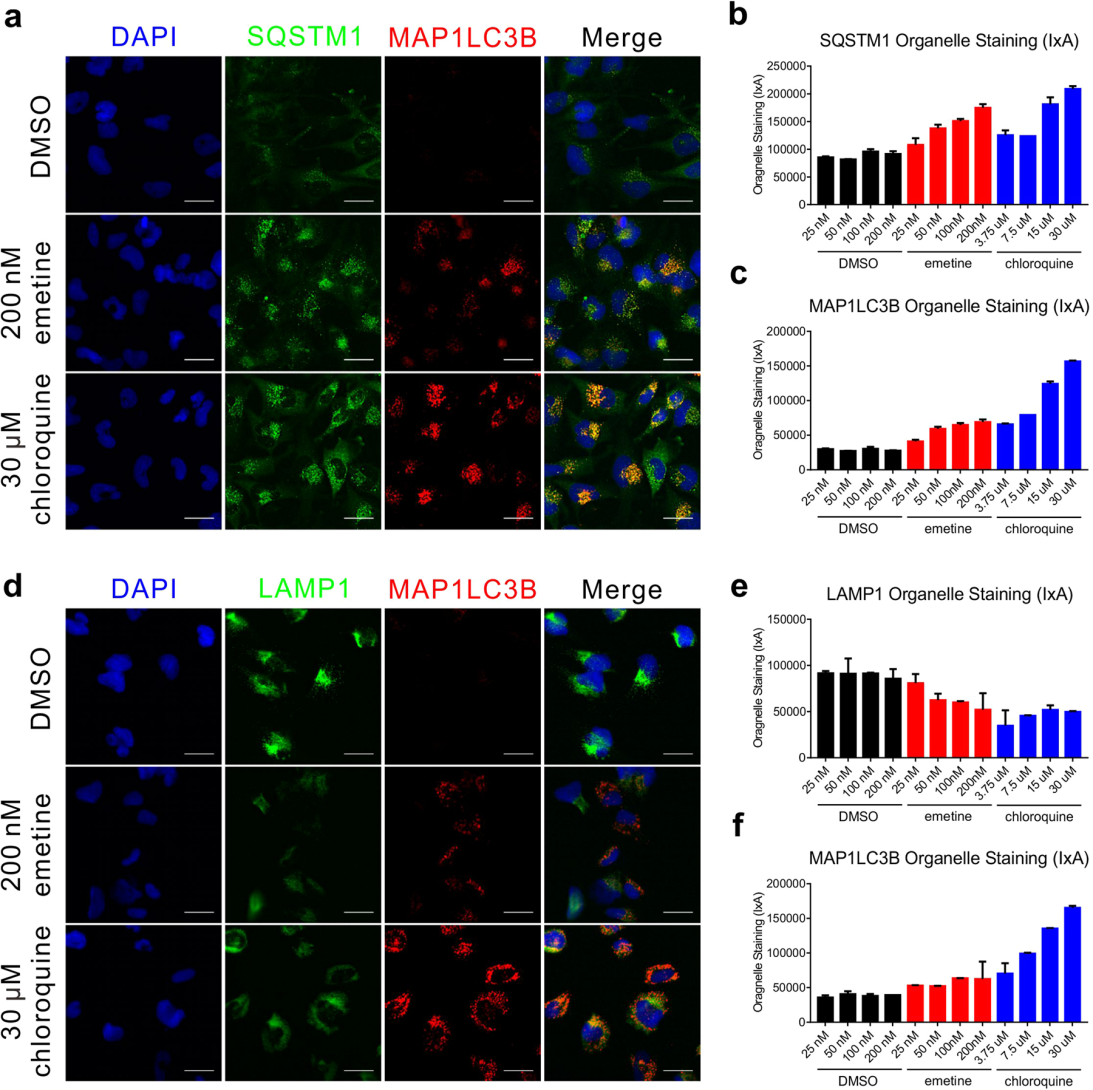
Emetine interrupts autophagy. **a** Immunofluorescent images of astrocytoma SNB-19 cells treated with DMSO, emetine or chloroquine for 24 h, then stained with SQSTM1 protein (green), MAP1LC3B protein (red) and nuclei (blue). **b** Quantitative analysis of the SQSTM1 puncta staining per cell (average puncta signal intensity per cell × average total puncta area per cell, I × A) in (**a**), showing the dose-dependent increase after emetine or chloroquine treatment. **c** Quantitative analysis of the MAP1LC3B puncta staining per cell (I × A) in (**a**), showing the dose-dependent increase after emetine or chloroquine treatment. **d** Immunofluorescent images of astrocytoma SNB-19 cells treated with DMSO, emetine or chloroquine for 24 h, then stained with LAMP1 protein (green), MAP1LC3B protein (red) and nuclei (blue). **e** Quantitative analysis of the LAMP1 vesicle staining per cell (I × A) in (**d**), showing the dose-dependent decrease after emetine or chloroquine treatment. **f** Quantitative analysis of the MAP1LC3B puncta staining per cell (IxA) in **d**, showing the dose-dependent increase after emetine or chloroquine treatment. Error bars indicate mean ± S.D. All values represent mean ± SD (*N* ≥ 3 replicates)

A recent publication reported that vertical ZIKV transmission is reduced by the autophagy inhibitor chloroquine^23^. Another report indicated that emetine inhibits autophagy^24^. As with chloroquine, immunostaining showed the accumulation of SQSTM1 and MAP1LC3B in emetine treated SNB-19 cells (Fig. 5a–c), indicating autophagy was blocked by emetine. Lysosomes are directly involved in autophagic flux by merging with autophagosomes to form autolysosomes. LAMP1, a lysosomal membrane resident protein, immunostaining revealed enlarged lysosomal structures in both emetine- and chloroquine-treated cells as compared to the smaller lysosomal puncta in the DMSO-treated cells (Fig. 5d–f). Collectively, these data indicated that emetine and cephaeline accumulated in lysosomes and interrupted lysosomal function leading to the inhibition of autophagy. This may have interfered with the autophagy-dependent virus infection pathway to prohibit ZIKV infection. Furthermore, autophagy inhibition impaired the cellular trafficking of cholesterol and other lipids, and resulted in their accumulation in the lysosome. This effectively suppressed the virus replication by limiting the free cholesterol available for virus particle assemble.

## Discussion

Emetine is a drug with pleotropic properties that has been utilized as both an anti-protozoal and an emetic^25–27^. It is derived from the ipecac root and takes its name from its emetic properties. The drug was prescribed for years in the treatment of intestinal amoebiasis and amoebic liver abscess, but its use was associated with side effects of nausea, vomiting, and in high doses cardiotoxic effects in a portion of the patients. The negative inotropic and chronotropic effects are mediated by blocking L-type calcium channels in the heart^28,29^. Dehydroemetine, a synthetic analog of the drug, was reported to retain its anti-amoebicidal properties while producing fewer side effects^30,31^.

Although there is limited knowledge on the use of emetine to treat amebic liver abscesses in pregnancy^32^, emetine is potentially toxic to the fetus and it is generally recommended to avoid of its use in pregnancy^33^. However, the use of emetine in pregnancy has been recorded^34^. An example to support emetine’s clinical use for pregnant women is Zidovudine (AZT), an anti-HIV drug approved in 1987 for non-pregnant adults and in 1994 for pregnant women after its toxicity to the fetus was evaluated^35,36^. Therefore, the benefit of emetine’s antiviral effect and its potential toxicity to women in the advanced stages of pregnancy should be considered for further evaluation because of its potential to treat of severe ZIKV infections associated with significant neurological complications in newborns.

It has been reported that emetine inhibits viral translation by blocking the 40S ribosomal protein S14 in host cells^37,38^. Studies have also indicated that emetine blocks HIV reverse transcriptase^39^, inhibits viral polymerases^9^, kills trypanosomes through DNA intercalation^27^, and suppresses human cytomegalovirus by disrupting the 40S ribosomal protein S14^26^. Furthermore, emetine has been shown to inhibit Dengue viral RNA synthesis^40^.

In this work, we demonstrated that emetine potently inhibited viral replication. We noted that the IC_50_ value in the cell-free RNA polymerase assay was 20-time higher (less potent) than in the cell-based viral infection assay. This may be due to the high local concentration of emetine inside cells so that the effective concentration is greater than in the cell-free NS5 RNA polymerase assay. Similar phenomena wherein emetine exhibited a 50-fold lower potency in a cell-free polymerase assay compared to a cell-based viral infection assay was previously reported^9^. Another possibility is that the cell-free enzyme assay with the recombinant enzyme may underestimate the real activity of emetine due the limitation of enzyme assays or an unfavorable conformation of the recombinant NS5 protein. We previously found that the inhibitory activities of glucocerebrosidase inhibitors determined by cell-free enzyme assay did not match those obtained from cell-based assays^41^.

We also found that emetine significantly increased filipin staining of unesterified cholesterol in lysosomes–a possible indication that the NPC1 cholesterol transporter function is affected. Interestingly, NPC1 is a reported EBOV-binding protein required for EBOV entry^18^ and HIV replication in vitro^42^. Although the IC_50_ values of emetine determined from filipin and Nile red staining assays were higher than those determined in the cell-based viral infection assay, the locally high compound concentration in lysosomes may explain its anti-EBOV viral entry activity in the live virus infection assay and its in vivo activity. Lipids are important cellular components for flavivirus infection, and the dose-dependent lipid accumulation suggests yet another potential mechanism by which emetine disrupts ZIKV infection^43^. Lysosomal malfunction can reduce autophagic flux and inhibit autophagy^44^, which may contribute to emetine’s effect. Thus, it is reasonable to propose NPC1 plays a role in emetine’s inhibitory effect on viral entry, albeit we do not have specific data to demonstrate emetine’s direct interaction with NPC1. Additional studies are needed to further elucidate the involvement of lysosomal protein function during ZIKV infection.

In general, antiviral drug design follows two development paths. The first path targets viruses by preventing viral entry and replication, RNA/DNA synthesis, and protein synthesis. The advantage of this approach is potentially identifying more selective compounds with fewer side effects, while the main disadvantage is the possibility of developing drug resistance due to rapid viral mutation of target DNA. The other path is to target host cell functions that are required for viral entry and replication. Viruses depend on receptor-mediated entry, and cellular machinery for virus nucleic acid synthesis, protein synthesis, viral assembly, and viral release. Targeting host cell function produces a lower probability of viral drug resistance because the compounds act on the host proteins. The disadvantage is the potential drug side effects in patients because of the possible target-mediated compound toxicity to host cells. Since anti-viral therapy typically has a short course of one to two weeks, the tolerability of compounds can be finely tuned during the drug development process. Emetine acts on the ZIKV NS5 polymerase, on the host cell’s lysosomal function, and 40S subunit of the ribosome. The multiple mechanisms of action result in a synergistic enhancement of emetine’s inhibition of ZIKV replication.

In conclusion, emetine is a potent inhibitor of ZIKV and EBOV infections, which acts on the ZIKV RdRp NS5, host cell’s lysosome, and 40S ribosomal subunit (reported previously). The synergistic effect of multiple targeting points contributes to its high potency and efficacy. The local high drug concentration in lysosomes increases emetine’s potency in cell-based assays and in vivo. In addition, this feature of emetine not only targets the viral polymerase directly but also targets host cellular proteins, making it difficult for the virus to develop a resistance to the drug. Based on these newly discovered mechanisms of action, emetine and cephaeline could be broad antiviral agents for the treatment of ZIKV, EBOV, and other viral infections. The drug design strategy of targeting multiple viral and host cell targets can be a new approach for designing and developing the next generation of antiviral agents.

## Materials and methods

### Cell culture and viruses

Vero E6 and Hela cells were maintained in Dulbecco’s Modified Eagle Medium (HyClone) (DMEM) supplemented with 10% fetal bovine serum (Sigma-Aldrich). The following Ebola viruses were used: Ebola virus NML/H.sapiens-lab/COD/1976/Mayinga-eGFP-p3 (EBOV/May-eGFP) (derived from an Ebola virus, family Filoviridae, genus Ebolavirus, species Zaire ebolavirus, GenBank accession No NC_002549), and mouse-adapted (MA) EBOV (Ebola virus USAMRIID/BALB/c-lab/COD/1976/Mayinga-MA-p3).

SNB-19 astrocytoma and HEK293 cells were maintained in RPMI-1640 Medium (ATCC) and Eagle’s Minimum Essential Medium (ATCC) (EMEM) Medium supplemented with 10% fetal bovine serum (Hyclone), respectively. The following viruses were used: ZIKV MR766 strain (Uganda, 1947), ZIKV PRVABC59 strain (Puerto Rico, 2015), ZIKV FSS13025 strain (Cambodia, 2010) and ZIKV^BR^ strain (Brazilian strain, Brazil-ZIKV2015).

### TR-FRET ZIKV NS-1 assay

HEK293 cells were cultured in EMEM medium (ATCC, Cat. # 30–2003) with 10% fetal bovine serum (GE healthcare Life Sciences, Cat. # SH30071.03), and 1% pen/strep (Gibco, Cat. # 15140–122). Cells were seeded in the 1536-well assay plate and incubated at 37 °C with 5% CO_2_ for 20 h. Compounds were added to cells and incubated for 1 h before addition of ZIKV solution to cells (MOI = 0.5). After an incubation at 37 °C for 24 h, the reagent mixture of TR-FRET ZIKV NS1 assay using two anti-ZIKV NS1 antibodies was added to assay plates (Lee et al., in preparation). Fluorescence signals were measured using an Envision plate reader (PerkinElmer). Data were normalized by using the cell-containing wells without ZIKV as a negative control (0% NS-1) and wells containing ZIKV infected cells as a positive control (100% NS-1 level). The percent inhibition of NS-1 with these small molecules was then calculated using Prism 7 (GraphPad, San Diego, CA).

### Western blot analysis

Cells were collected by trypsinization, pelleted and subsequent lysed in 1×Laemlli buffer. The lysates were then boiled, or the cells were directly lysed in 1×Laemlli buffer and boiled. Antibodies used were anti–ZIKV NS1 (1:2,000; BF-1225-36, BioFront Technologies, Tallahassee, FL) or anti-GAPDH (sc-25778, Santa Cruz Biotechnology, Texas).

### Viral titer by focus-forming unit (FFU) assay

ZIKV titers from cell supernatants were determined by infecting Vero cells for 48 h with a methylcellulose overlay and analyzing the plates for focus-forming units per ml (FFU/ml). Briefly, cell supernatant was titrated in triplicates onto a monolayer culture of Vero cells in 96-well plates and incubated at 37 °C for 2 h. Virus inoculum was removed and replaced with a methylcellulose overlay. Vero cells were incubated for an additional 48–72 h before fixation and incubated with anti–flavivirus group antigen overnight at 4 °C. The next day, fixed cells were washed three times with PBS and incubated with horseradish peroxidase (HRP)-conjugated anti-mouse secondary antibody for 1 h at room temperature, washed again three times with PBS and incubated with DAB peroxidase substrate for 10 min (Vector Labs).

### ZIKV RNA time-course

Vero E6 cells were plated one day prior to infection into 12-well plates. After overnight incubation, cells were pre-treated with 1 μM Emetine at 37 °C for 1 h prior to addition of ZIKV. Cells were then placed on ice, inoculated with ZIKV-PRVABC59 at MOI = 1, and incubated for 2 h on ice. Next, a viral inoculum was removed, and cells washed three times with 1 × phosphate buffered saline (PBS) to remove unbound virion. At this time point (0 h), cells were either collected by scraping and pelleting, or incubated at 37 °C and then later collected at each subsequent time-point by trypsinization and pelleting. Cell pellets were then processed for total RNA extraction using the Qiagen RNeasy Plus kit. Purified RNAs were then primed with random hexamers and reverse transcribed into cDNA using the SuperScript III First-Strand Synthesis kit according to manufacturer’s instructions (ThermoFisher). Quantitative PCR was performed using SYBR Green PCR master mix (Invitrogen), ZIKV specific primers (ZIKV-NS1-Forward: GGAGTTCAACTGACGGTCG; ZIKV-NS1-Reverse: TACCCCGAACCCATGATCCT) and cellular gene specific primers (GAPDH-Forward: TCACTGCCACCCAGAAGACTG; and GAPDH-Reverse: GGATGACCTTGCCCACAGC), and an Applied Biosystems 7500 Fast real-time PCR system. A 60–95 °C melt curve analysis following PCR was performed using default settings. Relative quantification was performed using the ΔΔ*C*_T_ method with GAPDH as the endogenous control, and the relative fold change was calculated by normalizing to the GAPDH levels in the control cells.

### Immunocytochemistry and determination of ZIKV infection rate

Cells were fixed with 4% paraformaldehyde (Sigma) for 15 min at room temperature. Samples were permeabilized with 0.25% Triton X-100 (Sigma) for 10 min and were blocked with cell staining buffer (BioLegend) for 1 h. Then, samples were incubated with Anti-Zika Envelope antibodies (BioFront Technologies) at 4 °C overnight, followed by multiple PBS washes and incubation with Anti-mouse IgG (H + L), F(ab’)2 Fragment (Alexa Fluor® 488 Conjugate) (Cell Signaling Technology) for 1 h at room temperature. Finally, nuclei were stained with 1 μg/ml Hoechst 33342 (Invitrogen) at room temperature for 15 min.

To determine the ZIKV infection rate, we took images using the IN Cell 2200 imaging system (GE Healthcare) with ×20 or ×40 objective lens.Imaging detection was performed using FITC (ENV + ) and DAPI (nucleus) filter sets. Image analysis was conducted using the IN Cell Analyzer software (Version 3.7.2). With a multi-target analysis protocol, nuclei were segmented using the top-hat segmentation method with a minimum area set at 50 μm^2^ and a sensitivity set to 0. ZIKV ENV staining was identified as “cells” by the analysis software and was segmented using the multi scale top-hat algorithm. Settings for ENV detection were set to 100 µm^2^ and a sensitivity setting of 0. A filter for ENV + cells was set to 800 fluorosence units based on visual inspection of several wells. Infected cells were those with an average intensity greater than 800 fluorescence units. For each field in each well, the ENV + cells were divided by the total cell number as determined by the nuclear staining and multiplied by 100% to calculate the infection rate per field. The final value is an average of three wells. Montages were generated using Fiji-ImageJ (NIH).

### Cellular thermal shift assay (CETSA)

CETSA was conducted, as previously described^45^. MR766 infected cells were harvested and rinsed with PBS, then re-suspended in detergent-free buffer (25 mM HEPES, pH 7.0, 20 mM MgCl2, 2 mM DTT) supplemented with protease inhibitors and phosphatase inhibitor cocktail. The cell suspensions were lysed via 3 freeze-thaw cycles with liquid nitrogen, followed by centrifugation at 20,000 × *g* for 20 min. at 4 °C to separate the soluble fraction from the cell debris. For the CETSA melting curve experiments, the cell lysates were diluted in detergent-free buffer and divided into two aliquots, followed by treatment with or without 50 μM emetine. After 60-min. treatment at room temperature, each sample was divided into 12 small aliquots in 50 μL/tube and heated individually at different temperatures (32–54 °C with 2 °C interval) for 3 min in a thermal cycler (Eppendorf), followed by immediate cooling for 3 min on ice. The heated cell extracts were centrifuged at 20,000 × *g* for 20 min. at 4 °C to separate the soluble fractions from precipitates. After centrifugation, the supernatant was analyzed by western blotting with anti-NS5 antibody (BF-8B8, BioFront Technologies, Tallahassee, FL). The relative chemiluminescence intensity of each sample at different temperatures was used to generate the temperature dependent melting curve. The apparent aggregation temperature (T_agg_) was calculated by nonlinear regression. The statistically significance between two curves were analyzed by extra sum-of-squares F test. All data represent mean ± SEM of at least 3 replicates.

### In vitro RNA polymerase assays

Recombinant ZIKV NS5 from MR766 ZIKV strain was expressed and purified in Dr. Heng Zhu’s lab. An RNA polymerase assay kit was purchased from Profoldin (Hudson, MA). RNA synthesis assays were performed in 10 µL of reactions following the manufacturer’s instructions. After 23 ng of purified Zika NS5 was added into 384-well small-volume plate in 3 µL, serial dilutions of emetine were added into the wells in 3 µL. The mixtures were pre-incubated for 30 mins at room temperature. A master mix containing single-stranded polyribonucleotide, 10 µM of NTP mix, 20 mM Tris–HCl, pH 8.0, 1 mM DTT, and 8 mM MgCl_2_ was added into each well in 4 µL. The reactions were incubated at 37 °C for 1 h and then stopped by adding the fluorescence dye in 10 µL. The fluorescence intensities (Ex = 485 ± 5, Em = 535 ± 10 nm) were measured using a Tecan plate reader (Tecan).

### Molecular docking and modeling of emetine bound to ZIKV NS5 RNA-polymerase

Crystal structure of the ZIKV NS5 RdRp was retrieved from Protein Data Base (PDB) (5U0B). Sequence alignment and structural comparison of the ZIKV RdRp with Dengue virus, hepatitis C virus (HCV), and Ebola was analyzed. Docking of emetine to ZIKV NS5 RdRp domain was performed using the MOE dock program. The ligand induced fit protocol was applied and the binding affinities of docked poses were evaluated using the GBVI/WSA score. The best binding model with the lowest binding free energies was further energetically-minimized using the MOE program.

### Ebola VLP beta-lactamase entry assays

Ebola VLPs containing a beta-lactamase-fused VP40 protein (EBOV BlaVP40) and GP were produced in Dr. García-Sastre’s lab, as previously described^46^. LiveBLAzer FRET–B/G Loading Kit with CCF2-AM and Opti-MEM reduced serum medium were purchased from Life Technologies (Carlsbad, CA, USA). The Ebola VLP assay were performed, as previously described^47^. Briefly, HeLa cells were plated at 750 cells/well in 3 µL; 23 nL of the drug solution was transferred to the assay plate. The cells were treated with 1 µL/well VLP. The CCF2-AM beta-lactamase substrate was added and fluorescence intensities were measured using an Envision plate reader. The assay was run in triplicate. High throughput drug screening in Ebola VLP assay using HeLa cells was conducted similarly, as previously described^7^.

### Emetine and cephaeline inhibit EBOV infection in vitro

The inhibition assay was performed as described previously^48^. In brief, Vero E6 cells were pre-treated with emetine or cephaeline (0–2.0 µM) or DMEM alone for 1 h at 37 °C, and infected with a MOI = 0.1 of GFP-expressing EBOV in the presence of emetine, cephaeline or DMEM alone for 1 h at 37 °C. Cells were then further incubated for 72 h in the presence of Emetine, Cephaeline or DMEM. At 72 h, the green fluorescent protein signal was quantified on a Biotek Synergy HTX plate reader. Infection was determined by comparing fluorescence readings of emetine or cephaeline-treated infected cells to DMEM-treated controls. The EC_50_ and EC_90_ values were calculated using a four-parameter logistic regression in Prism 5 (GraphPad).

### Filipin staining

WT fibroblast cells from a human male GM05659 (Coriell institution) were seeded in black with a clear bottom tissue culture-treated 96-well plates. After overnight incubation, cells were treated with a serially diluted compound and incubated for 24 h. Cells were incubated with 8 μM Nuclear Green^TM^ LCS1 (AAT Bioquest), diluted in medium, at 37 °C for 15 min to stain the nucleus. After washing twice with PBS, cells were fixed with 100 μl/well 4% paraformaldehyde (Sigma) at room temperature for 15 min. Then, cells were stained with 100 μl/well 50 ng/ml filipin (Sigma), freshly dissolved in DMSO at 10 mg/ml and then diluted in PBS, at room temperature for 1 h. The images were taken by the IN Cell2200 automated fluorescence plate imaging reader (GE Healthcare) with ×20 or ×40 objective lens, and imaging detection was performed using DAPI (filipin) and FITC (nucleus) filter sets. Montages were generated using Fiji-ImageJ (NIH).

### LysoTracker dye staining

The cells were cultured and treated as described above. On the day of the experiment, cells were live-stained with 100 μl/well of 50 nM LysoTracker Red DND-99 dye (Invitrogen) in the medium at 37 °C for 1 h, followed by fixation in 100 μl/well 4% paraformaldehyde (Sigma) for 15 min. and twice washes with PBS. The nuclear staining was performed by an addition of 100 μl/well of 1 μg/ml Hoechst 33342 (Invitrogen) in PBS and incubation at room temperature for 15 min. The images were taken by the IN Cell 2200 automated fluorescence plate imaging reader (GE Healthcare) with ×20 or ×40 objective lens, and imaging detection was performed using DAPI (nucleus) and TRITC (LysoTracker) filter sets. Montages were generated using Fiji-ImageJ (NIH).

### Nile red staining

The cells were cultured and treated, as described above. On the day of the experiment, cells were live-stained with 1 μM Nile red dye, prepared in cell culture medium, and incubated at 37 °C for 10 min. After washing twice with PBS, the cells were fixed in 4% paraformaldehyde (Sigma) in PBS at 100 μl/well for 15 min at room temperature. The nuclear staining was carried out by addition of 1 μg/ml Hoechst 33342 (Invitrogen) in PBS and incubation at room temperature for 15 min. The images were taken by the IN Cell2200 automated fluorescence plate imaging reader (GE Healthcare) with ×20 or ×40 objective lens, and imaging detection was performed using DAPI (nucleus) and Cy3 (lipid droplet) filter sets. Montages were generated using Fiji-ImageJ (NIH).

### Immunostaining

Cells were fixed with 100% methanol for 10 min at −30 °C. Cells were blocked with Cell Staining Buffer for 1 h at room temperature. The cells were incubated overnight at 4 °C with the following primary antibodies in Cell Staining Buffer: rabbit anti-Lamp1, mouse anti-p62, rabbit anti-LC3B. On the following day, the cells were washed three times with PBS and incubated with secondary antibodies goat anti-mouse Alexa Fluor 488 and goat anti-rabbit Alexa Fluor 488. Cells were then washed with PBS, stained with nuclear dye Hoechst 33342 and incubated for 30 min in the dark at room temperature. After a final wash in PBS, 100 µL of fresh PBS was added to the cells for imaging on the INCell 2500 HS (GE Healthcare, Port Washington, NY. The INCell Developer software was used for high-content image analysis. Statistical analysis and graphs were generated using Prism GraphPad 7 (San Diego, CA). Montages were generated using Fiji-ImageJ (NIH).

### Ethical statement

The animal work involved with EBOV was approved by the Animal Care Committee (ACC) at the Canadian Science Center for Human and Animal Health (CSCHAH) and performed in accordance with the guidelines from the Canadian Council on Animal Care (CCAC). Studies involving live, infectious Ebola virus were conducted under biosafety level 4 (BSL-4) conditions at the National Microbiology Laboratory located in Winnipeg, Canada.

The in vivo efficacy study in Ifnar1^−/−^ mice was approved by the Institutional Animal Care and Use Committee of Florida State University and in accordance with Public Health Service Policy and the Guide for the Care and Use of Laboratory Animals (8th edition).

Animal procedures with SJL mice and ZIKV were performed in agreement with the Public Health Service Policy on Humane Care and Use of Laboratory Animals (United States) and with the approval of the Sanford Burnham Prebys Medical Discovery Institute Institutional Animal Care and Use Committee, protocol AUF# 16-049. Studies involving live Zika virus were conducted under biosafety level 2 + (BSL-2 + ) conditions at the Sanford Burnham Prebys Medical Discovery Institute located in La Jolla, California, USA.

### ZIKV^BR^ infection of SJL mice and Real-time qPCR quantification

Three-month-old SJL male mice (Jackson Laboratories, Bar Harbor, ME, USA) were infected retro-orbitally with ZIKV^BR^ (1 × 10^5^ PFU in 0.2 ml PBS; Brazilian strain, Brazil-ZKIV2015). To titer ZIKV^BR^ in the blood, the blood samples (0.1 ml each) were collected at two days post infection. After that emetine (1 mg/kg/day) was delivered to SJL male mice (four mice per group) via IP for the next 6 days. At the same time the group of SJL mice (*n* = 4) received the same volume of VC buffer. On day 7 the blood samples were collected and then animals were killed.

Total RNA was extracted from the blood samples (0.1 ml each) using a NucleoSpin RNA Kit (Macherey-Nagel) according to manufacturer instructions. RNA concentration was measured using a NanoDrop spectrophotometer (NanoDrop Technologies). The samples were kept at −80 °C until use. ZIKV-specific primers (ZIKV-835 5′-TTGGTCATGATACTGCTGATTGC-3′ and ZIKV-911c 5′-CCTTCCACAAAGTCCCTATTGC-3′) were described previously^49,50^. Real-time qPCR was performed using a QuantiTect Reverse Transcription Kit (QIAGEN), SYBR Green I Master Mix, and a LightCycler 480 II instrument (Roche) using the following conditions: initiation at 95 °C for 10 min. followed by 50 cycles of 95 °C for 15 s, 60°C for 30 s, and 72 °C for 30 s. Data were analyzed using LightCycler 480 Software 1.5.0 (Roche). Assay sensitivity was determined using samples with the known ZIKV^BR^ concentrations. GraphPad Prism 5 was used as fitting software.

### Viremia profiling in Ifnar 1^−/−^ mice

Ifnar1^−/−^ mice (5–6 week old, *n* = 4) were infected with FSS13025 strain of ZIKV (1 × 10^3^ FFU/in 250 µl of EMEM media/mouse) IP. Blood samples were taken from the tail vein at 24 h intervals until 5 d.p.i. NS1 levels were quantified in the serum using ZIKV NS1 ELSA kit. On day 0, the sample was taken just prior to infection.

### ZIKV infection in Ifnar1^−/−^ mice

Ifnar1^−/−^ mice (6–7 weeks old, male and female) were dosed daily with Emetine intraperitoneally (IP) starting with 24 h prior and 24 h after challenge with 1 × 10^3^ FFU/mouse FSS13025-ZIKV day 0. The control group with vehicle control (PBS) and ZIKV were also maintained. Treatment continued until day 3. Mice were killed on day 3, and blood and liver tissue immediately harvested. Serum viral loads were analyzed using ZIKV-NS1 sandwich ELISA kit (BioFront Technologies). From the liver tissue stored in RNAlater (Qiagen), the total RNA was isolated using RNeasy mini kit (Qiagen) and viral load were estimated by real time PCR using ZIKV-NS1 specific primers and GAPDH primers as described above in the methods for ZIKV RNA time course.

### Evaluating the protective efficacy of emetine and cephaeline against MA-EBOV in mice

Six to eight week-old BALB/C mice (Charles River), female, were randomly assigned into groups (6 per group). All the mice were challenged with a dose of 1000 times the lethal dose (LD_50_) MA- EBOV via IP. Treatments with either emetine (1 mg/kg/day) or cephaeline (5 mg/kg/day) or PBS (same volume) for the control group were initiated at 3 h prior to challenge and continued for up to 6 d.p.i. All animals were monitored for signs of disease and weight change for 14  days post challenge, and survival for additional 14 days.

### Data and statistical analysis

The primary screen data were analyzed using customized software developed internally^51^. All data were presented as mean ± SEM with at least three independent experiments unless otherwise stated. Half maximal inhibitory concentration (IC_50_) of compounds was calculated using Prism software (GraphPad Software, San Diego, CA). All imaging data are presented as mean ± SD and represent data from cells in at least 10 fields from three or more independent experiments. The two-tailed unpaired Student’s test of the mean was used for single comparisons of statistical significance between experimental groups. However, one-way analysis of variance (ANOVA) with a Bonferroni test was used for multiple comparisons.

### Data availability

All data and protocols are available upon request.

## Electronic supplementary material

Emetine inhibits Zika and Ebola
