# Supplementary material for: Emetine inhibits Zika and Ebola virus infections through two molecular mechanisms: inhibiting viral replication and decreasing viral entry
Source: Cell Discov. 2018 Jun 5;4:31. doi: 10.1038/s41421-018-0034-1 (PMC5986771; doi:10.1038/s41421-018-0034-1)
Supplement: Supplementary file 1 — Emetine inhibits Zika and Ebola [file 41421_2018_34_MOESM1_ESM.pdf]

Emetine inhibits Zika and Ebola virus infections through two molecular mechanisms: inhibiting viral replication and decreasing viral entry

Shu Yang[1,11], Miao Xu[1,11], Emily M Lee[2,11], Kirill Gorshkov[1], Sergey A. Shiryaev[4], Shihua He[5], Wei Sun[1], Yu-San Cheng[1], Xin Hu[1], Anil Mathew Tharappel[2], Billy Lu[1], Antonella Pinto[4], Chen Farhy[4], Chun-Teng Huang[4], Zirui Zhang[5], Wenjun Zhu[5], Yuying Wu[3], Yi Zhou[3], Guang Song[7], Heng Zhu[7], Khalida Shamim[1], Carles Martínez-Romero[8], Adolfo García-Sastre[8], Richard A. Preston[9], Dushyantha T. Jayaweera[9], Ruili Huang[1], Wenwei Huang[1], Menghang Xia[1], Anton Simeonov[1], Guoli Ming[10], Xiangguo Qiu[5,6,*], Alexey V. Terskikh[4,*], Hengli Tang[2,*], Hongjun Song[10,*], Wei Zheng[1,*]

[1]National Center for Advancing Translational Sciences, National Institutes of Health, 9800 Medical Center Drive, Bethesda MD 20892, USA.

[2]Department of Biological Science, Florida State University, Tallahassee, Florida, 32306, USA.

[3]Department of Biomedical Sciences, Florida State University, Tallahassee, Florida, 32306, USA.

[4]Development, Aging and Regeneration Program, Sanford Burnham Prebys Medical Discovery Institute, La Jolla, CA 92037, USA.

[5]Special Pathogens Program, National Microbiology Laboratory, Public Health Agency of Canada, Winnipeg, Manitoba R3E 3R2, Canada.

[6]Department of Medical Microbiology, University of Manitoba, Winnipeg, Manitoba R3E 0J9, Canada.

[7]Department of Pharmacology & Molecular Sciences; Johns Hopkins School of Medicine, Baltimore, MD, USA.

[8]Department of Microbiology and Global Health and Emerging Pathogens Institute, Icahn School of Medicine at Mount Sinai, New York, NY 10029, USA.

[9]Department of Medicine, Miller School of Medicine, University of Miami, Miami FL 33136, USA.

[10]Department of Neuroscience and Mahoney Institute for Neurosciences, University of Pennsylvania, Philadelphia, PA 19104, USA.

[11]These authors contributed equally to this work.

[12]These authors jointly directed this work.

[12]Correspondence should be addressed to X.Q. (Xiangguo.qiu@canada.ca), A.V.T. (terskikh@sbpdiscovery.org), H.T. (tang@bio.fsu.edu), H.S. (shongju1@jhmi.edu), W. Zheng. (wzheng@mail.nih.gov).

Running title: Emetine inhibits Zika virus and Ebola virus infections

Keywords: Zika virus, Ebola virus, emetine, cephaeline, viral replication, viral entry, lysosomal function

Supplementary Figure S1

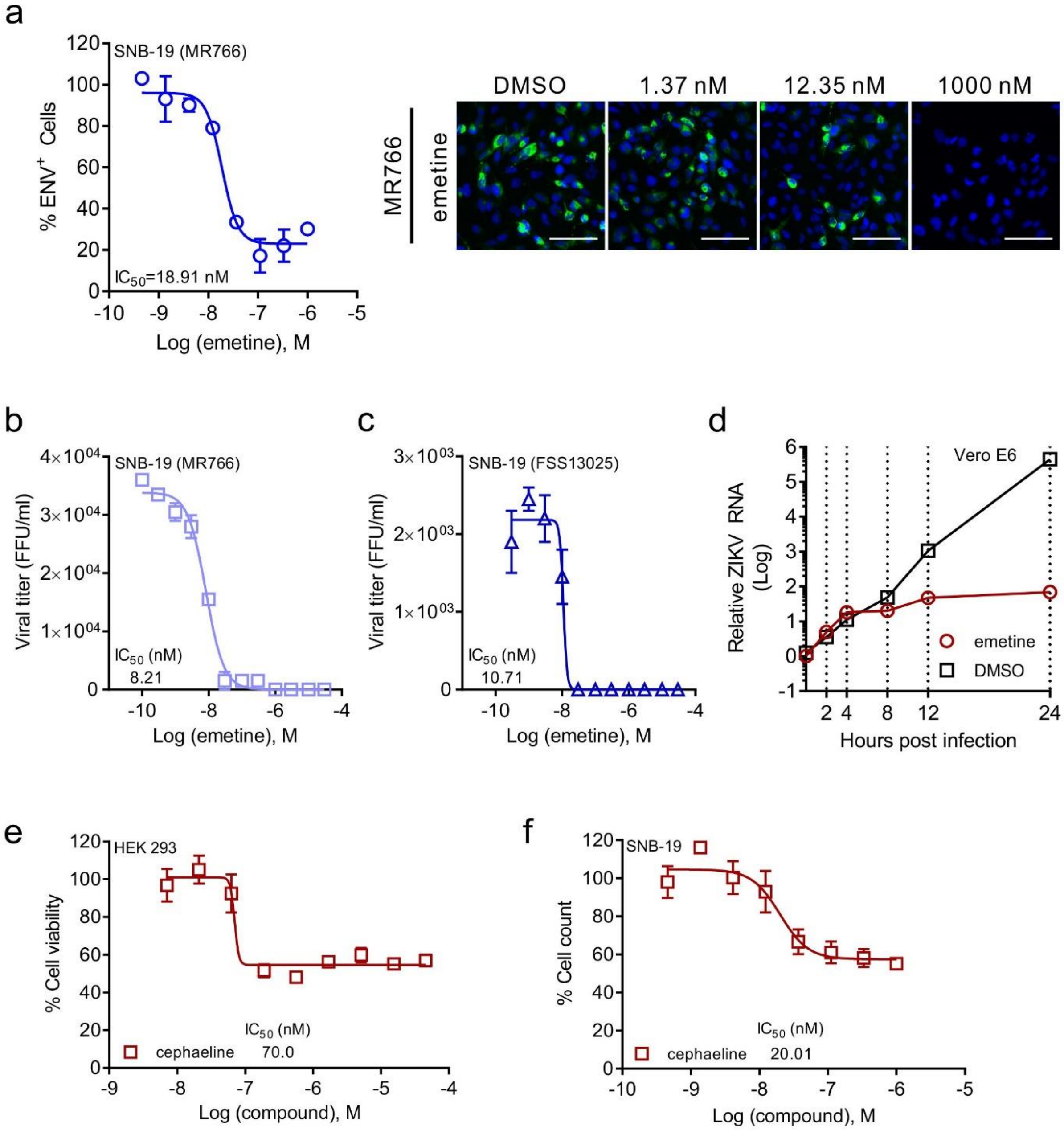

Supplementary Figure S1. Emetine suppresses ZIKV infection in SNB-19 and Vero E6 cells. (**a**) Left, dose-response curves showing the inhibition effect of emetine on ZIKV (strain MR766 or strain PRVABC59) infection measured by envelope protein positive (ENV+) astrocytoma cells. Right, immunofluorescent images of astrocytoma cells from a stained for ZIKV ENV protein (green) and nuclei (blue) and treated with emetine. (**b**) Virus production from MR766-infected

Vero E6 cells treated with emetine. (**c**) Virus production from Zika strain FSS13025-infected Vero E6 cells treated with emetine. (**d**) ZIKV RNA quantification from Vero E6 cells treated with emetine or DMSO after virus infection. (**e**) Dose-response curves showing HEK293 cell viability after treatment with cephaeline measured by ATPLite assay. (**f**) Dose-response curves showing astrocytoma cell number after cephaeline treatment measured by nuclear staining. All values represent mean ± SD (n = 3 replicates). All curves represent best fits for calculating the IC_{50} values (graph inset). Scale bar, 100 μm.

Supplementary Figure S2

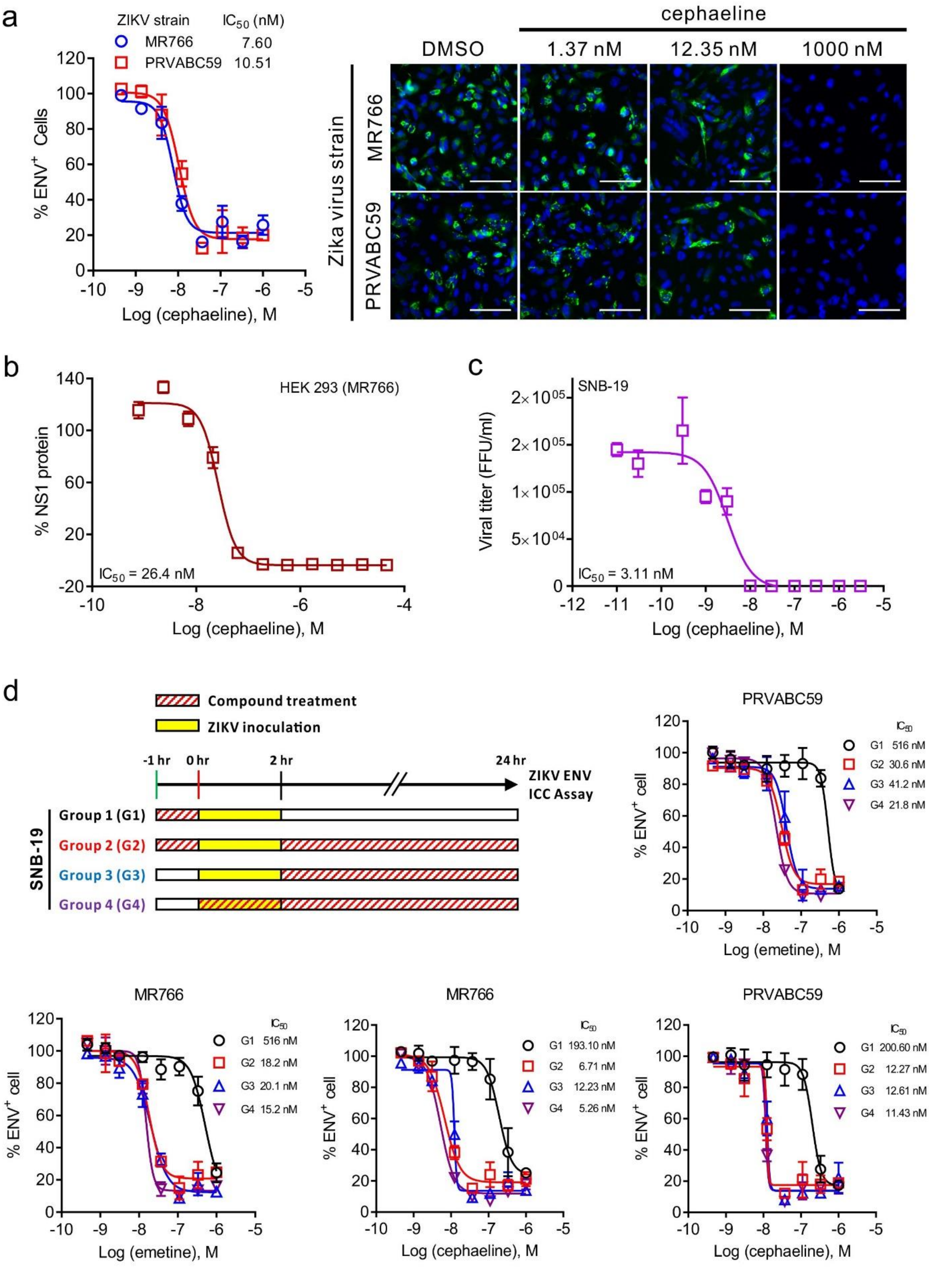

Supplementary Figure S2. Emetine and cephaeline are inhibitors of ZIKV infection and replication. (**a**) Left, dose-response curves showing the inhibition effect of cephaeline on ZIKV MR766 and PRVABC59 infection measured by ENV+ astrocytoma cells, respectively. Right, immunofluorescent images of astrocytoma cells from a stained for ZIKV ENV protein (green) and nuclei (blue) and treated with cephaeline. (**b**) Dose-response curves showing the inhibition effect of cephaeline treatment on ZIKV NS1 protein expression in HEK293 cells exposed to ZIKV MR766 strain. (**c**) Virus production from cephaeline-treated SNB-19 cells. (**d**) Experimental design for four different treatment regimens in astrocytoma cells. In the timeline, green bar indicates first addition of DMSO (white rectangle) or compound (red striped rectangle) one hour prior to washout and ZIKV inoculation (yellow rectangle) at time 0 h (red bar). Black bar at time 2 h indicates washout and addition of DMSO or compound. Total time for assay was 24 h. (right and bottom) Normalized log dose-response curves showing the inhibition effect of emetine or cephaeline on ZIKV PRVABC59 infection measured by ENV+ astrocytoma cells using four different treatment regimens. All values represent mean ± SD (n = 3 replicates). All curves represent best fits for calculating the IC_{50} values (graph inset). Scale bar, 100 µm.

Supplementary Figure S3

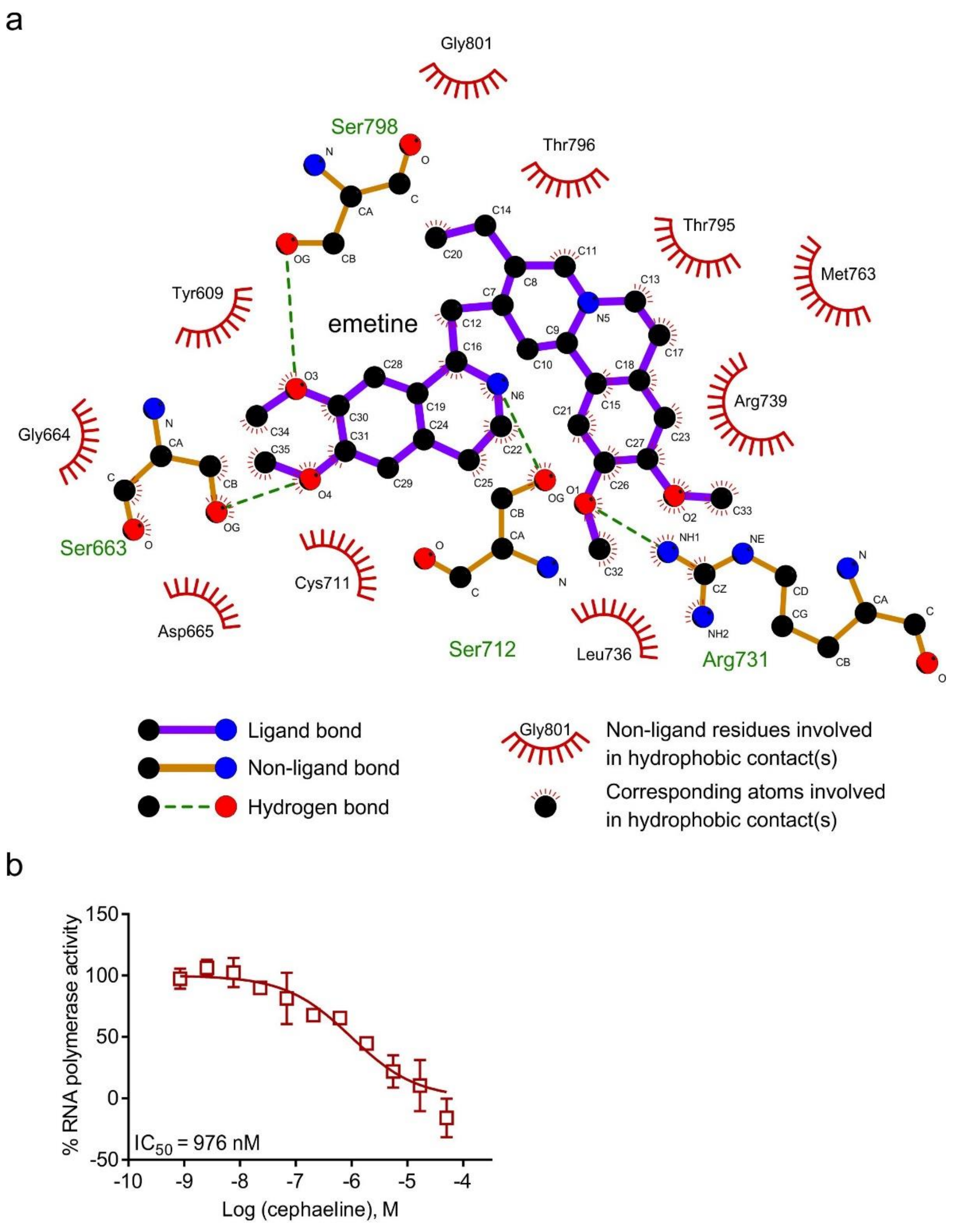

Supplementary Figures S3. Emetine inhibits ZIKV NS5 polymerase activity. (**a**) Detail binding site information of the virtual docking results of the Zika NS5-Emetine complex. (**b**) Dose-response curve showing the inhibition effect of cephaeline treatment on the RNA polymerase activity of recombinant Zika NS5 enzyme by the cell free assay. All values represent mean ± SD (n=3 replicates). All curves represent best fits for calculating the IC_{50} values (graph inset).

Supplementary Figure S4

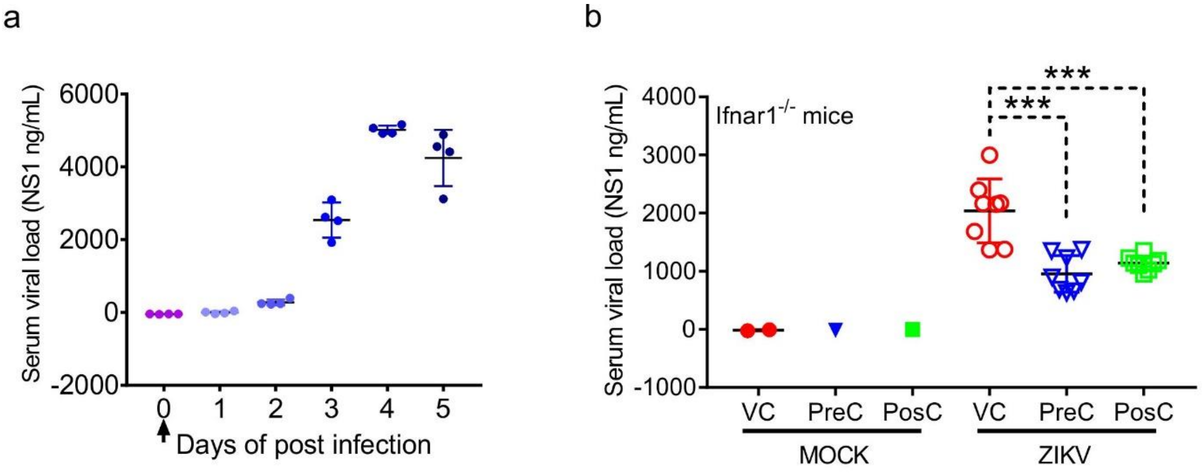

Supplementary Figure S4. Emetine suppressed ZIKV load *in vivo.* (**a**) Viremia profile of Ifnar1$^{-/-}$ mice. Interferon (alpha and beta) receptor 1 null (Ifnar1$^{-/-}$) mice (5-6 week old, n=4) were infected with FSS13025 strain of ZIKV (1X10^{3} FFU/in 250 µl of EMEM media/mouse) IP. Blood samples were taken from the tail vein at 24 hour intervals until 5 d.p.i. NS1 levels were quantified in the serum using ZIKV NS1 ELSA kit. On day 0, the sample was taken just prior to infection. (**b**) Ifnar1$^{-/-}$ mice (8-9 week old, male and female) were dosed with Cephaeline 2 mg/kg/day IP starting at 24 hour prior (PreC, n=8) or 24 hour after (PosC, n=8) challenging with ZIKV on day 0. Drug was continued until day 3. Mice were euthanized and blood was collected. NS1 protein in the serum was estimated using ZIKV-NS1 ELISA kit. Values presented as mean ± SEM, one way ANOVA, followed by Dunnett's test. ***p<0.001

Supplementary Figure S5

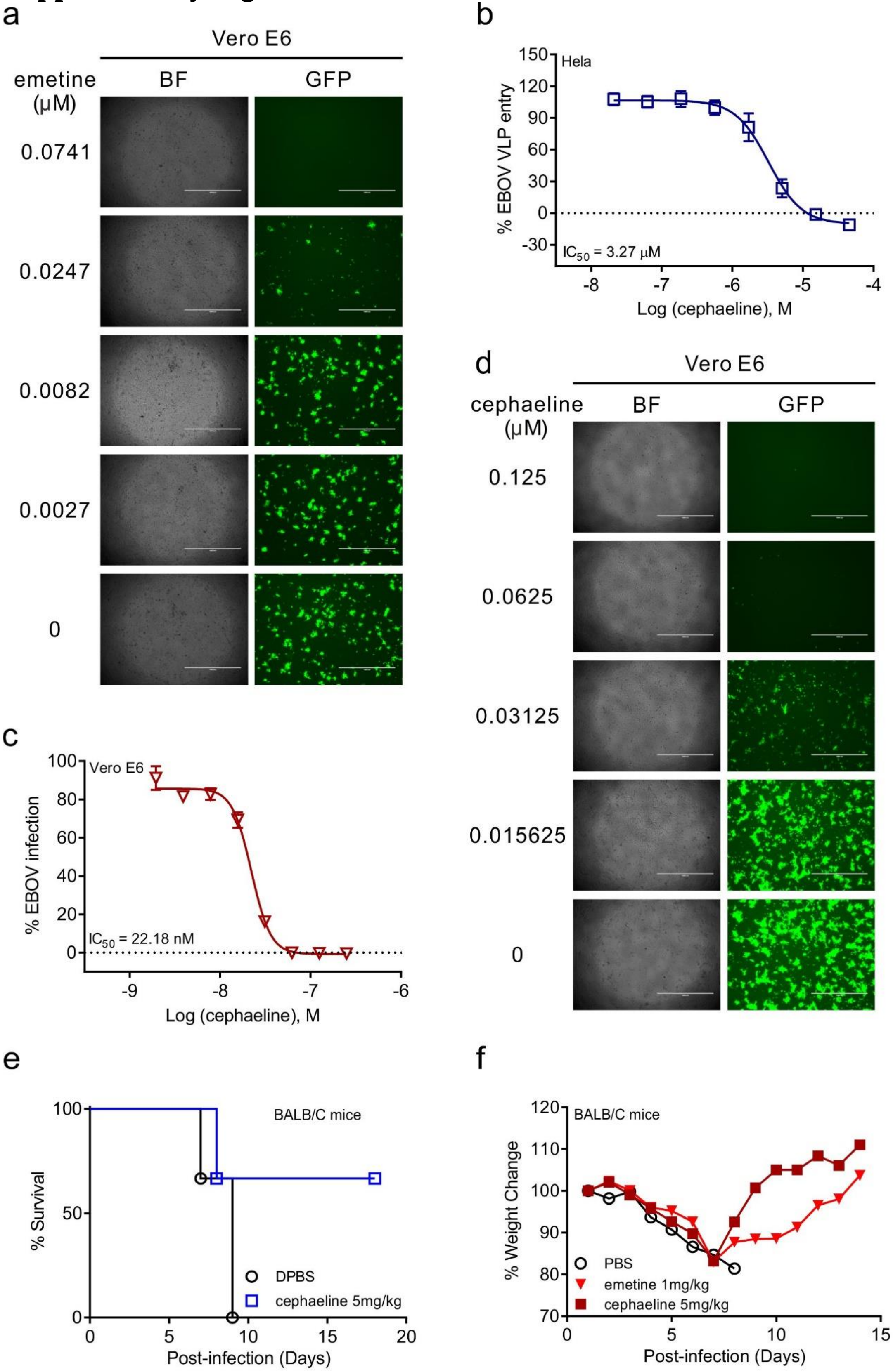

Supplementary Figure S5. Emetine and cephaeline suppress EBOV infection *in vitro and in vivo*. (**a**) Bright field (BF) and fluorescent GFP images of Vero E6 cells treated with emetine and infected with EBOV/eGFP virus. (**b**) Dose-response curves showing the inhibition effect of cephaeline treatment on Ebola VLP entry in HeLa cells. (**c**) Dose-response curves showing the inhibition effect of cephaeline treatment on infection of Ebola live virus in Vero E6 cells. (**d**) Bright field (BF) and fluorescent GFP images of Vero E6 cells treated with cephaeline and infected with EBOV/eGFP virus. Bright field and fluorescent GFP images were captured. (**e**) The survival curve of MA-EBOV infected mouse treated with 5 mg/kg cephaeline every day. Six- to eight-week-old female Balb/C mice were randomly assigned into groups (n=6 animals). All the mice were challenged with a lethal dose of 1000 X the LD_{50} mouse adapted EBOV via IP. Treatments with either cephaeline (5mg/kg/day) or PBS (same volume for the control group) were initiated at 3 h before challenge and continued for up to 6 days post infection. Survival was monitored for 28 days post infection. (**f**) Weight change of EBOV infected-mice (n = 6 mice) treated with 1 mg/kg/day emetine and 5 mg/kg/day cephaeline, respectively. All values represent mean ± SD (n = 3 replicates). All curves represent best fits for calculating the IC_{50} values.

Supplementary Figure S6

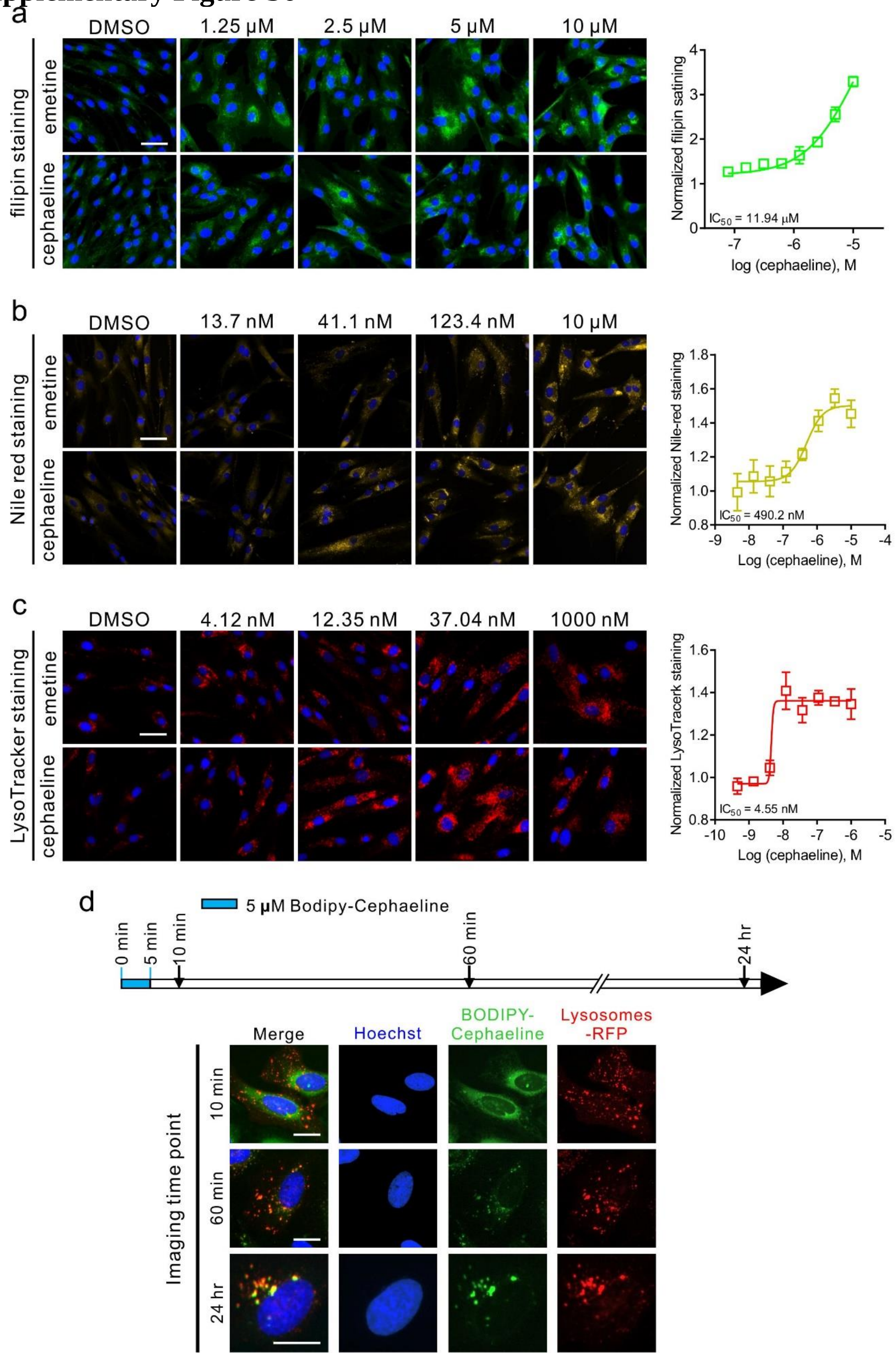

Supplementary Figure S6. Emetine induces lysosome accumulation. (**a**) Left, fluorescence images of fibroblast cells treated with emetine or cephaeline and stained with filipin (unesterified cholesterol, green) and nuclear green (nuclei, blue). Scale bar, 50 µm. Right, dose-response curve of cells in **a** showing the effect of cephaeline on cholesterol accumulation measured by Filipin dye fluorescence intensity. (**b**) Left, fluorescence images of fibroblast cells treated with emetine or cephaeline and stained with LysoTracker dye (acidic organelles, red) and Hoechst 33442 (nuclei, blue). Scale bar, 50 µm. Right, dose-response curve of cells in **b** showing the effect of cephaeline on acidic organelle accumulation measured by Lysotracker dye fluorescence intensity. **(c)** Left, fluorescence images of fibroblast cells treated with emetine or cephaeline and stained with Nile red (lipids, yellow) and Hoechst 33442 (nuclei, blue). Scale bar, 50 µm. Right, normalized Log dose-response curve of cells in **c** showing the effect of cephaeline on lipid accumulation measured by Nile red fluorescence intensity. (**d**) BODIPY-cephaeline tracks the position of the emetine analog in cells after treatment. In the timeline, blue bar indicates addition of BODIPY-cephaeline 5 min. prior to washout. Black bar at time 10 min., 60 min. and 24 h indicates the imaging time-point. Bottom, fluorescence images of U2OS cells treated with BODIPY-cephaeline and stained with CellLight™ Lysosomes-RFP, BacMam 2.0 (lysosome, red) and Hoechst 33442 (nuclei, blue). Scale bar, 20 µm. All values represent mean ± SD (n=3 replicates). All curves represent best fits for calculating the IC_{50} values (graph inset).
